# Supplementary material for: Multi‐dataset identification of innovative feature genes and molecular mechanisms in keratoconus
Source: J Cell Mol Med. 2024 Sep 19;28(18):e70079. doi: 10.1111/jcmm.70079 (PMC11412914; doi:10.1111/jcmm.70079)
Supplement: Supplementary file 2 — Material S2. [file JCMM-28-e70079-s002.zip › 7.GSEA analysis/6.SNORA74B/KEGG_PORPHYRIN_AND_CHLOROPHYLL_METABOLISM.html]

Details for gene set KEGG\_PORPHYRIN\_AND\_CHLOROPHYLL\_METABOLISM[GSEA]

|  || Dataset | SNORA74B.group.cls#h\_versus\_l |
| Phenotype | group.cls#h\_versus\_l |
| Upregulated in class | h |
| GeneSet | KEGG\_PORPHYRIN\_AND\_CHLOROPHYLL\_METABOLISM |
| Enrichment Score (ES) | 0.3280701 |
| Normalized Enrichment Score (NES) | 1.0568774 |
| Nominal p-value | 0.40881765 |
| FDR q-value | 1.0 |
| FWER p-Value | 0.998 |
Table: GSEA Results Summary

  

Fig 1: Enrichment plot: KEGG\_PORPHYRIN\_AND\_CHLOROPHYLL\_METABOLISM      
 Profile of the Running ES Score & Positions of GeneSet Members on the Rank Ordered List

  

| SYMBOL | TITLE | RANK IN GENE LIST | RANK METRIC SCORE | RUNNING ES | CORE ENRICHMENT || 1 | UGT1A8 | na | 321 | 0.680 | 0.1587 | Yes |
| 2 | UGT1A6 | na | 2066 | 0.366 | 0.1987 | Yes |
| 3 | ALAS2 | na | 3264 | 0.288 | 0.2353 | Yes |
| 4 | UGT2B4 | na | 3661 | 0.272 | 0.2911 | Yes |
| 5 | UGT2B11 | na | 6088 | 0.190 | 0.2680 | Yes |
| 6 | MMAB | na | 7459 | 0.149 | 0.2655 | Yes |
| 7 | UGT2B28 | na | 7492 | 0.149 | 0.3013 | Yes |
| 8 | ALAD | na | 7776 | 0.142 | 0.3281 | Yes |
| 9 | UGT2B10 | na | 9771 | 0.097 | 0.2946 | No |
| 10 | CPOX | na | 13351 | 0.043 | 0.2021 | No |
| 11 | PPOX | na | 13384 | 0.043 | 0.2118 | No |
| 12 | UGT2A3 | na | 13387 | 0.043 | 0.2224 | No |
| 13 | HMOX1 | na | 14464 | 0.031 | 0.1989 | No |
| 14 | FECH | na | 16285 | 0.012 | 0.1493 | No |
| 15 | BLVRB | na | 16909 | 0.005 | 0.1327 | No |
| 16 | HMOX2 | na | 17296 | 0.002 | 0.1220 | No |
| 17 | UROD | na | 17344 | 0.001 | 0.1209 | No |
| 18 | FTH1 | na | 18115 | -0.007 | 0.1005 | No |
| 19 | HMBS | na | 18942 | -0.016 | 0.0807 | No |
| 20 | EARS2 | na | 19226 | -0.020 | 0.0775 | No |
| 21 | GUSB | na | 19513 | -0.023 | 0.0750 | No |
| 22 | BLVRA | na | 19579 | -0.024 | 0.0790 | No |
| 23 | UROS | na | 19606 | -0.024 | 0.0842 | No |
| 24 | UGT2A1 | na | 21014 | -0.040 | 0.0535 | No |
| 25 | HCCS | na | 22991 | -0.065 | 0.0127 | No |
| 26 | COX15 | na | 23210 | -0.068 | 0.0233 | No |
| 27 | FTMT | na | 25011 | -0.099 | -0.0042 | No |
| 28 | UGT2B15 | na | 25363 | -0.105 | 0.0115 | No |
| 29 | ALAS1 | na | 25821 | -0.113 | 0.0263 | No |
| 30 | COX10 | na | 29335 | -0.204 | -0.0245 | No |
| 31 | UGT2B7 | na | 31877 | -0.316 | -0.0196 | No |
| 32 | CP | na | 33180 | -0.412 | 0.0446 | No |
Table: GSEA details [plain text format]

  

Fig 2: KEGG\_PORPHYRIN\_AND\_CHLOROPHYLL\_METABOLISM      
 Blue-Pink O' Gram in the Space of the Analyzed GeneSet

  

Fig 3: KEGG\_PORPHYRIN\_AND\_CHLOROPHYLL\_METABOLISM: Random ES distribution      
 Gene set null distribution of ES for **KEGG\_PORPHYRIN\_AND\_CHLOROPHYLL\_METABOLISM**
